# Foraging Behavior of Goats Browsing in Southern Mediterranean Forest Rangeland

**DOI:** 10.3390/ani10020196

**Published:** 2020-01-23

**Authors:** Youssef Chebli, Samira El Otmani, Mouad Chentouf, Jean-Luc Hornick, Jérôme Bindelle, Jean-François Cabaraux

**Affiliations:** 1 Department of Veterinary Management of Animal Resources, University of Liège, Avenue de Cureghem 6, B43, 4000 Liège, Belgium; selotmani@doct.uliege.be (S.E.O.); jlhornick@ulg.ac.be (J.-L.H.); jfcabaraux@uliege.be (J.-F.C.); 2 National Institute of Agricultural Research (INRA), 78 Bd. Mohamed Ben Abdellah, Tangier 90010, Morocco; mouad.chentouf@gmail.com; 3 Precision Livestock and Nutrition Unit, Gembloux Agro-Bio Tech, University of Liège, Passage des Déportés 2, 5030 Gembloux, Belgium; jerome.bindelle@uliege.be

**Keywords:** goat, feeding behavior, forage availability, diet composition, Northern Morocco

## Abstract

**Simple Summary:**

Grazing goats in forests is an ancestral practice in the Mediterranean region. This study aims to assess the seasonal variations in the feeding behavior of goats browsing in the Mediterranean forest rangeland of Northern Morocco for two years. The goats’ diet was largely composed of woody species. Overall, the smaller the bite mass, the higher the biting rate, leading to an increased instantaneous intake rate. During the dry season, goats tend to compensate for the low intake rate by extending daily grazing time, thus reducing the sensitivity of intake rates to forage availability. A particular high selection of cork oak was observed over seasons. The higher diet diversity was recorded during summer and fall compared to the spring. Nevertheless, it should be remembered that the diet selection of goats is ultimately influenced by the herder’s decisions. Results confirm the high adaptability of goats to the seasonality of complex Moroccan forest rangelands.

**Abstract:**

Mediterranean forest rangelands offer an important feed source for goats. Concerns about grazing strategies and management schemes in order to ensure the rangeland sustainability of Southern Mediterranean forest have revived interest in the foraging behavior of goats. This study was conducted to investigate the seasonal changes of feeding behavior of grazing goats in the Southern Mediterranean forest rangeland of Northern Morocco during two consecutive years beginning in 2016. The direct observation method was used to compare diet composition, intake rate, and diet selectivity of goats during three seasons (spring, summer, and fall). Bite mass of each plant species selected by goats was estimated using hand-plucked simulation. The optimal foraging theory was used as a tool to explain the goats foraging decisions. Bite mass range was extremely wide and varied seasonally. The goats’ diet was largely composed of *Cistus* spp., *Lavandula stoechas*, *Quercus* spp., and *Myrtus communis*. The result shows that the smaller the bite mass, the higher the biting rate, leading to increased short term intake rates. The selection of various plant species during fall and summer enlarged the diet diversity of goats. As expected, goats preferred trees and some shrubs despite their low availability. Consequently, the most available species is not necessarily the most positively selected. Particular high and positive selection of *Quercus suber* was observed over seasons. The outcomes confirm the high adaptability and ability of goats to select a woody species across seasons. Knowledge about forage availability and the feeding behavior of goats could be used as the first guide for rangeland managers to ensure herd and forest sustainability.

## 1. Introduction

Domestic goats (*Capra hircus*) have been associated with mankind for more than 10,000 years [1] and have grazed Mediterranean forests for millennia [2]. Overall, goats have an important multifunctional role in marginal habitats and have always been considered a useful and specialized ruminant browsing Mediterranean forest rangelands [3]. However, in the case of low forage availability and overgrazing, they could also be viewed as a problem for forest regeneration [4,5]. They have a very efficient selective foraging behavior and the ability to thrive better in harsh environments. Based on these criteria, goats are qualified as “opportunistic feeders” [6].

Moroccan forest rangelands yield 1.5 billion feed units per year, corresponding to 80% of the feed requirements of grazing animals. The herds browsing in Moroccan forest rangelands are about 32% of the national herd, i.e., approximatively 8 million heads. In the Mediterranean region, Morocco occupies the third position in terms of goat population with 5.2 million heads [7].

Northern Morocco is characterized by the coexistence of two livestock systems [8]. The first system is an agropastoral system based on sheep and cattle reared inside the farms (<10 heads). The second livestock system, which is the dominant one, is the traditional extensive system, based on grazing forest rangelands, where the herd is composed only of goats. The mountainous topography, including the existing forest vegetation, and animal adaptation could explain the predominance of goats in the North Moroccan woodlands. Overall, these goats are not supplemented because of the high prices of feed supplementation, the poverty of goats’ herders, and the presence of forest rangelands that remain free from grazing fees [9]. The goat population is about 627,000 heads localized in mountainous and isolated areas of the region [10]. The average size of goat flocks is less than 80 animals per farm. The number of goats fluctuates throughout the years depending on drought periods and herder motivation [11]. Generally, goats are reared for meat production intended for the traditional local market. The annual productivity of goat herding system is characterized by a low gross margin, compared to the same goat system in the northern part of the Mediterranean area [8]. Despite this, goat farming plays an important socio-economic role and contributes approximately from 68% to 100% of farmer incomes [8].

Understanding the goats’ preferences for specific plant species and plant parts and how such preferences and selective behavior vary along the grazing season is a key factor to consider when developing grazing strategies and management schemes that enhance the sustainable exploitation of the grazed vegetation.

In the Mediterranean region, the large body of studies about the feeding behavior and diet composition of goats in forest rangelands were mainly conducted in the Northern [12,13,14] and Eastern countries [15,16,17,18]. Limited information is available for the Southern shore of the Mediterranean, which shares similar agro-climatic conditions with the other parts of the Mediterranean but displays specific socio-economic features that impact how grazed herds are managed. Only a small portion of the Southwestern Argan (*Argania spinosa*) forest of Morocco (Atlantic region) have benefited from research efforts on the importance of the Argan tree in goat feeding [19]. Due to the lack of information on the seasonal foraging behavior of goats, it is still difficult to develop grazing strategies and management schemes in order to ensure a sustainable forest rangeland exploitation combined with adequate foraging of the grazing animals.

This study was therefore undertaken to determine diet composition, intake rate, selectivity, and diet diversity of goats in the Southern Mediterranean forest rangeland of Northern Morocco over three seasons of two consecutive years.

## 2. Materials and Methods

### 2.1. Study area description

**Figure 1:**
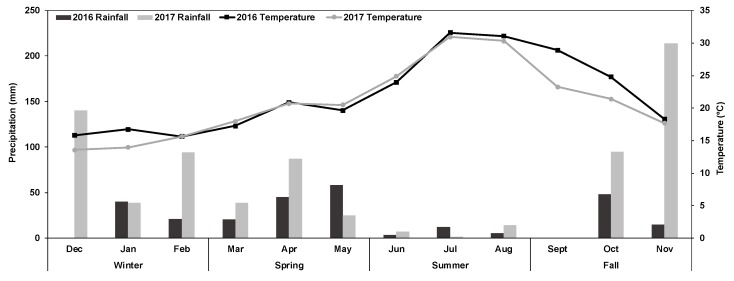
Monthly mean of air temperature (°C) and precipitation (mm) of 2016 and 2017 in Chefchaouen (Northern Morocco). Data source: DRATT [10].

The study was carried out for two consecutive years in the Rif region of Northern Morocco. The climate is of Mediterranean type, characterized by seasonal contrast, pre-humid in mountainous areas (rainy and cold in winter and mild in summer), and humid in winter and dry in summer in the plain. Mean annual rainfall is around 700 mm, with a daily temperature range of 3–14 °C (minimum) and 18–38 °C (maximum) [9]. The two experimental years were very contrasted regarding the mean annual rainfall, with 270 and 755 mm in 2016 (dry year) and 2017 (wet year), respectively (Figure 1).

### 2.2. Experimental Pasture and Goat Management

The study was conducted in the forest rangeland of Chefchaouen (5°08’ N; 5°18’ W; 1195 to 1250 m a.s.l) during three seasons (spring, summer, and fall) of 2016 and 2017. This rangeland is a domanial forest covered with heterogeneous vegetation that goes from the low formations of the rockrose species (*Cistus* spp.), resulting from the degradation of the sylvatic series, up to the high oak groves. Vegetation includes oak species (*Quercus* spp.), inclusive of Algerian (*Q. canariensis)*, holm (*Q. ilex)*, and cork (*Q. suber*) oaks associated with shrublands dominated by the strawberry tree (*Arbutus unedo* L.); and the *Cistus* spp., inclusive of wrinkle-leaved (*C. crispus*), Montpellier (*C. monspeliensis*), and sage-leaved (*C. salviifolius*) rockroses [9,20].

Grazing in the forest is practiced during spring, summer, and fall under the supervision of the herder himself or a family member [9]. For the winter, the duration of grazing decreases to values as low as 1 to 3 hours per day (browsing fallow land around the farm), which explains the exclusion of this season from the study. During this season, pasture access is very limited, herders delimb tree branches as fodder and bring them to the goat shed [9,21]. Livestock watering is guaranteed by water sources and streams inside the grazed forested rangeland.

### 2.3. Forage Availability

The seasonal forage availability of plant species consumed by goats is required to calculate diet selectivity [22,23]. For biomass measurements, several numbers and sizes of quadrats were tested to get the most representative quadrat to minimize the effect of rangeland heterogeneity. Therefore, forty quadrats of 40 m² (4 × 10 m) were implemented seasonally in the rangeland. The measurements were undertaken in the last month of each studied season (May, August, and November). The non-destructive method known as the reference module was used for shrubs and trees, as described by Chebli et al. [24]. For trees, we considered only the accessible and consumed plant parts for goats (height <1.5 m), which are small-sized trees damaged by delimbing [9]. For herbaceous species (mostly grass), forty quadrats of 1 m² were installed, each one embedded within one shrub quadrat. A destructive method was used, where all herbaceous biomass was cut. Biomass samples were oven-dried at 55 °C to constant weight to obtain the dry matter (DM).

### 2.4. Familiarization Procedure

The animal familiarization procedure aims to accustom the flock to the permanent presence of an unfamiliar person. This mutual familiarization procedure was detailed by Bonnet et al. and Meuret and Provenza [25,26]. The observer is considered fully familiarized with the animal if he can get as close as 0.5 to 1.5 m, providing that it does not hamper the spontaneous movements of the goats [27,28,29]. As reported by Perevolotsky et al. [30], grazing behavior is not affected by the observer’s presence after a familiarization period. A three-day familiarization period for observers was necessary to accustom the flock to their presence. The success of the familiarization procedure makes precise bite counts and consumed plant identification possible.

### 2.5. Plant Identification, Direct Observation, and Bite Mass Simulation

The direct observation method was used to estimate the bite number and botanical composition of goat diets for three consecutive grazing days during three seasons. Eight alpine goats from a flock of 72 adult goats, with similar physical conditions of 42 ± 2.5 kg live weight and an average age of 36 ± 6 months, were selected for this study. The flock was chosen among the most representative in the study area, in addition to the voluntary desire of the herder to cooperate throughout the study period. The herder managed the grazing time and circuits by himself. For the experimentation, after consulting the procedures described by several authors [12,13,14,15,19,28], continuous bite observations were performed over the entire grazing days. Data were collected over 10-min snapshots by focal-animal sampling, each goat being observed thrice per day (morning, mid-day and afternoon). The same goats were observed every day and season during both years. Observers recorded the botanic composition and the number (*n*) of bites on each consumed plant species that allowed generating the total number of bites (*TB*). The percentage of bites per plant species (*TB_i_*, %) was calculated using the following equation:

TB_i_ = NB_i_/TB, (1)

where *NB_i_* is the number of observed bites of plant *i*. Bite mass (*BM*, g DM/bite), i.e., the average mass of hand-plucked simulation of each consumed forage by the animal, as described by Cook [31], was measured. One hundred hand-plucked simulations per consumed part of plant species were collected separately in paper bags, dried in an oven at 40 °C to constant weight, and weighed to obtain the average mass dry matter per bite.

BM = hand-plucked samples/100.(2)

The observation and hand plucked simulation of bites were performed during each season. The average intake rate (*IR*, g DM/min) was expressed as

IR = BR × BM,(3)

where *BR* is the biting rate (*BR*, n/min).

Diet composition (*DC*, %) was reported as the percentage of each consumed species in the diet according to the following equation:

(4) $$\mathrm{DC}\;=\frac{{\mathrm{NB}}_{i}{\;\times \;\mathrm{BM}}_{i}}{\sum_{i=1}^{n}\left(\mathrm{NB}\;\times \;\mathrm{BM}\right)}\;,\;$$

where *NB_i_* is the number of observed bites of plant *i*, *BM_i_* is the mean bite mass of the plant *i*, and *n* the number of plant species (*n* =16).

To understand the foraging behavior of goats, some foraging decisions were briefly noted during the observation procedure. The optimal foraging theory (OFT) is used as a tool to discuss these decisions [32].

### 2.6. Diet Measurement Index

#### 2.6.1. Diet Diversity

Diet diversity was calculated through Levins’ diversity index, also called diet breadth [33]. As suggested by Hurlbert [34], the diversity index is standardized to express it on a scale from 0 to 1, following measurement for Levins’ standardized diversity index (SDI):

(5) $$\mathrm{SDI}=\frac{\left(\frac{1}{\sum_{i=1}^{n}r_{i}{}^{2}}\right)-1\;}{n-1},$$

where *r_i_* is the proportion of plant *i* in the diet, and *n* the number of plants (*n* = 16).

#### 2.6.2. Diet Selectivity

Diet selectivity is calculated through Ivlev’s index of selectivity (SI) [35]. It is widely used as a mean of comparing feeding habits with the availability of potential feed resources in natural habitats. The purpose of this index is to characterize the degree of selection of a particular plant species by an animal. The relationship is defined as

(6) $$\mathrm{SI}=\frac{r_{i}-p_{i}}{r_{i}+p_{i}}\;,$$

where *r_i_* is the proportion of plant *i* in the diet, and *P_i_* is the proportion availability of plant *i* in the rangeland. The index has a possible range of −1 to +1, the negative values for the rejected part of plant species, zero for random selection, and positive values for active selection [22].

#### 2.6.3. Diet Overlap

Diet overlap (similarity) of goats was compared between seasons and years using the Morisita–Horn index [36]; it is considered the least biased overlap index [37]. Index values range from zero (no overlap) to one (complete overlap). The formula is as below:

(7) $$C_{H}=\frac{2\sum_{i}^{n}P_{\mathrm{ij}}P_{\mathrm{ik}}}{\sum_{i}^{n}P^{2}{}_{\mathrm{ij}}+\;\sum_{i}^{n}P^{2}{}_{\mathrm{ik}}}\;,$$

where *C_H_* is the diet overlap, *P_ij_* is the proportion of the diet in season *i* of the total proportion of the diet in year *j*, *P_ik_* is the proportion of the diet in season *i* of the total proportion of the diet in year *k*, and *n* is the total number of seasons (*n* = 3).

### 2.7. Statistical Analysis

Bite mass was analyzed using a three-way analysis of variance (ANOVA) with factors plant species, season, and year. Before analysis, data expressed in percentage were arcsine-square root-transformed to normalize the distribution [38]. Foraging behavior data were analyzed using the PROC MIXED procedure of SAS [39] with “day × goat” as the experimental unit (days = 3, goats = 8). The model contained the fixed variable season (i.e., spring, summer, and fall) and year (i.e., 2016 and 2017) and their interactions. Goat was considered as a random effect to prevent this variance from being incorporated in the error term of the analysis. For all data, the random statement specified the covariance structure “CS” (compound symmetry), chosen by the lower “AIC” (Akaike’s information criteria) among other structures. For all analyses, the significance level was declared at *p* < 0.05.

## 3. Results

### 3.1. Forage Availability

**Figure 2:**
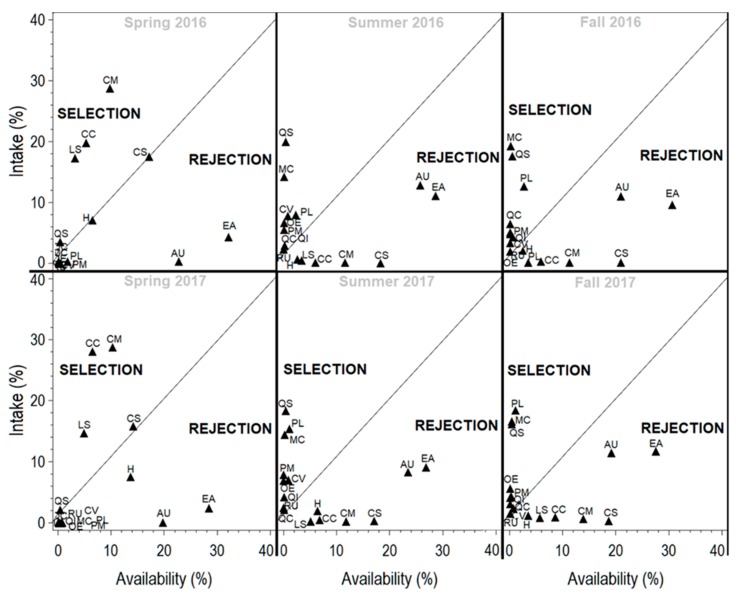
Seasonal selection vs. rejection of plant species consumed by goats browsing a Southern Mediterranean forest rangeland during 2016 and 2017. AU: *Arbutus unedo;* CC: *Cistus crispus;* CM: *Cistus monspeliensis;* CS: *Cistus salviifolius;* CV: *Calicotome villosa;* EA: *Erica arborea;* H: Herbaceous; LS: *Lavandula stoechas;* MC: *Myrtus communis;* OE: *Olea europaea;* PL: *Pistacia lentiscus;* PM: *Phillyrea media;* QC: *Quercus canariensis*; QI: *Quercus ilex;* QS: *Quercus suber;* RU: *Rubus ulmifolius*.

The study area was covered by heterogeneous vegetation composed mainly of three distinct groups of plant species: herbaceous (mainly grass and forbs) shrubs (*Arbutus unedo* L.; spiny broom (*Calicotome villosa* (Poir.) Link); *Cistus* spp.; tree heath (*Erica arborea* L.); topped lavender (*Lavandula stoechas* L.); common myrtle (*Myrtus communis* L.); broad-leaved phillyrea (*Phillyrea media* L.); lentisk (*Pistacia lentiscus* L.); elm-leaf blackberry (*Rubus ulmifolius* Schott.); and trees (*Quercus canariensis* L., *Quercus ilex* L., *Quercus suber* L.; and European olive (*Olea europaea* L.: *O. europaea* var. *sylvestris* (Mill) Lehr). Based on our direct observations and discussion with herders, these listed plant species are the main dietary components of goats. Forage availability was affected by the season, the year and their interaction (*p* < 0.01) (Table 1). The results indicated a higher forage availability during spring compared to the fall and summer of both studied years. The seasonal change of feeding behavior (intake rate) in terms of forage availability of each plant species is displayed in Figure 2.

### 3.2. Bite Mass

The bite mass of each plant varied significantly by season (*p* < 0.001), year (*p* < 0.001) and their interaction (*p* < 0.05), except for *E. arborea* and *P. media*, which were not significantly affected by year and the interaction of season and year, respectively (Table 2). Moreover, bite mass varied significantly (*p* < 0.001) among individual plant species.

Bite mass of *Cistus spp*., *E. arborea*, herbaceous, and *L. stoechas* were significantly larger in spring, ranging from 0.193 to 0.339 g DM/bite in 2016 and from 0.223 to 0.430 g DM/bite in 2017, respectively. However, their bite mass was smaller (<0.185 DM/bite) during the fall of 2016 and the summer of 2017. The opposite trend was observed for the rest of plant species, whose bite mass were larger in the fall and summer, varying from 0.089 to 0.239 g DM/bite and from 0.118 to 0.341 g DM/bite in 2016 and 2017, respectively, while in spring of both years, bite mass recorded smaller values (<0.245 g DM/bite). For all plant species, the bite mass recorded in 2017 was higher than those of 2016, except for *R. ulmifolius* in spring and *E. arborea* in summer.

### 3.3. Diet Composition

The composition of the diet was significantly affected by season (*p* < 0.001) (Table 3). No significant differences (*p* > 0.05) were observed between years concerning the contribution of *C. monspeliensis*, *C. salviifolius*, *E. arborea*, herbaceous, *L. stoechas*, and *P. media*. In the same trend, the diet contribution of *C. monspeliensis*, *Q. ilex*, and *Q. suber* were not significantly affected by the interaction between seasons and years.

During the spring of 2016, the contribution of *Cistus spp.* was the highest with 66%, followed by *L. stoechas* (17.3%), and herbaceous (7%). These species contributed lowly to the diet during fall and summer (<3%). The diet proportion of *R. ulmifolius* was the lowest with 0.01%. In the fall and summer, the diet proportion of *Quercus spp*., *M. communis*, *P. lentiscus*, *A. unedo*, and *E. arborea* was largely significant.

The same list of plant species in 2016 was consumed during each season of 2017. In comparison with 2016, during spring, the contribution of *C. crispus* was significantly increased by 42% with the decreased rate of *C. salviifolius* and *L. stoechas* by 10% and 15%, respectively. In the fall, the greatest increase in contribution to the diet was observed for *O. europea* followed by *P. lentiscus*, and *E. arborea*. The opposite trend was observed with the diet proportion of *Q. canariensis* and *C. villosa*. In summer, the contribution of *P. lentiscus* and *P. media* was increased by 93% and 17%, respectively. On the other hand, the diet contribution of *A. unedo* and *E. arborea* was decreased by 35% and 17%, respectively.

On average, the diet of the goats was largely composed of shrubs (64% to 90%) and trees (2% to 35%). However, the contribution of herbaceous did not exceed 8%. The contribution of trees to the diet during spring dropped from 30.3% to 3.7% and from 29.0% to 2.2% in 2016 and 2017, respectively.

### 3.4. Biting and Intake Rate

Season (*p* < 0.001), year (*p* < 0.01), and their interaction (*p* < 0.05) significantly affected the average bite rate. The higher values were recorded during the fall with 22.3 and 20.81 bites/min in 2016 and 2017, respectively (Table 1).

Season affected (*p* < 0.001) the total bites of each consumed plant species by goats (Table 4). The same trend (*p* < 0.05) was found in the year except for *C. salviifolius*, *E. arborea*, herbaceous, *L. stoechas*, and *P. media*. The interaction effects between season and year were also significant except for *C. salviifolius* and *Q. ilex*. The highest number of bites was recorded for *Cistus spp*., herbaceous plants, and *L. stoechas* in spring and the lowest number in the fall and summer of both years. The opposite trend was observed for the rest of the consumed plant species. The higher and lower values of total bites per consumed plant species were observed during the spring of both years. Thus, the higher percentage of bites was recorded for *C. crispus* with 26.5% in 2017 and the lower percentage (<0.16%) for *C. villosa*, *Q. canariensis,* and *R. ulmifolius* during both years.

The average intake rate was significantly affected by the season (*p* < 0.001) of each studied year. Intake rate was higher during the spring with 4.41 and 5.10 g DM/min in 2016 and 2017, respectively (Table 1). The lower values were recorded during the fall and summer of both years, varying from 3.21 to 4.25 g DM/min. The interaction between seasons and years had not a significant effect on the average intake rate (*p* > 0.05).

Regardless of the low availability of some species such as *C. villosa*, *Quercus spp.*, *M. communis*, and *P. lentiscus*, they were highly consumed by goats mainly in the fall and summer of both years, as displayed in Figure 2. *Cistus* spp. and *L. stoechas* were ingested proportionally to their abundance only during the spring. Despite the high availability of *A. unedo* and *E. arborea*, they were avoided during all seasons.

### 3.5. Diet Diversity, Selectivity, and Overlap

The diet diversity of goats was significantly affected by season, year, and their interaction (*p* < 0.05). The higher diet diversity was recorded in the fall and summer of both years in which their values were significantly similar. The lower diet diversity was observed in spring with a value of 0.27 and 0.21 in 2016 and 2017, respectively (Table 1).

The season had a significant effect on the individual plant selectivity index (*p* < 0.01) during both years (Table 5). The same trend was observed for the effect of year (*p* < 0.05) except for *C. salviifolius* and *P. lentiscus*. The interaction between season and year had not a significant effect (*p* > 0.05) for *C. salviifolius*, *Q. canariensis*, and *Q. ilex*. The *Q. suber* was positively selected during all seasons (0.01 to 1). Similarly, *M. communis* was positively selected (from 0.4 to 1), except in the spring of 2017 (−0.66). *Cistus spp*. and *L. stoechas* were negatively selected in the all year-season (from –0.70 to −1) except during the spring of both years.

The results indicate a very high diet overlap of goats for the same season across years (from 0.77 to 1) and between fall and summer. The spring diet was the one that differed the most from the other seasons (from 0.05 to 0.12) (Table 6).

## 4. Discussion

### 4.1. Forage Availability 

Seasonal forage availability can be explained by the growing conditions of each plant favored, mainly by precipitation recorded during winter, early spring, and late fall (Figure 1). During the dry season, the considerable decrease in forage availability is provoked by water stress combined with high air temperature, interrupting and even ending the growth cycle of most plant species, especially annuals. The lower rainfall recorded in 2016 compared to 2017 might explain the inter-annual variability of feed resource availability. Similarly, several studies conducted in Mediterranean forest rangeland confirmed the primary reliance of forage availability on rainfall and air temperature and declines of forage availability during summer and fall are usually observed in similar studies [24,40,41]. Seasonal variations of forage availability were also confirmed by Salt et al. and Butt et al. [42,43].

### 4.2. Foraging Behavior Decisions of Goats

According to Papachristou et al. [44], the bulk of small ruminant diet includes few woody and herbaceous species, representing less than ten species. 

Bite mass and biting rate are considered as key factors in the process governing the constitution of the daily diet of grazing animals, especially on heterogeneous rangelands [16,30].

The bite mass ranges of different consumed parts of plant species were extremely wide. Similarly, Manousidis et al. [14] found a very large range of bite mass for local Greek goats (0.042 to 0.972 g DM) browsing in Northern Mediterranean woody rangelands. In forested rangelands of Southern France, dominated by *Q. pubescens*, bite mass of alpine goats varied from 0.88 to 1.68 g DM [12]. These results are much higher than those found by Fomum et al. [45], who estimated the bite mass of Nguni goats ranged from 0.10 to 0.60 g DM in a South African woodland.

As reported in this study, the findings of Manousidis et al. [14] confirmed the inter-annual variability of diet composition. In the same way, other studies have stated the seasonality of diet composition, such as in the central Monte desert of Argentina [46] and Northern Mediterranean forest [47].

The average biting rate were approximatively similar to those reported by Meuret [48] and Fomum et al. [45] for alpine goats in Northern Mediterranean woodland (8–30 bites/min) and for Nguni goats in South African rangelands (9–22 bites/min), respectively.

In the present study, the average intake rate ranged from 3.21 to 5.10 g DM/min. Similar seasonal and yearly changes in the average intake rate were reported by Manousidis et al. [14] in Northern Mediterranean woodland (2.83–5.65 g DM/min).

According to our direct observations, due to the low forage availability in the summer and fall, goats spent more time moving between feeding stations to maximize their instantaneous intake rate, in line with the Optimal Foraging Theory (OFT) that explains instantaneous decisions of foraging herbivores with regards to energy and time trade-offs of the grazing process [32,49,50,51]. Indeed, as noted by Charnov [32], the reduced forage availability causes the reduced time spent by animals at each feeding station and, consequently, conducts an increase in traveling duration spent between feeding stations and patches. Utsumi et al. [52] reported that the increasing distance between feeding stations decreased the average intake rate. It also could be assumed that goats make decisions to maximize their instantaneous intake rate during a foraging bout by increasing their biting rate or by mostly selecting plants with a large bite mass. The intake rate variation is related to the seasonal variations in both biting rate and bite mass. Our result shows that the smaller the bite mass, the higher the biting rate, through a possible compensatory mechanism to maintain short term intake rates. It is consistent with previous findings that show that animals must display compensatory mechanisms [14,19]; increasing the biting rate is one of these mechanisms.

Bite mass increases with the availability of each plant species in the pasture and, consequently, the intake rate when selectivity increases. As reported by Ungar and Noy-Meir [49], the sensitivity of the intake rate to variations of biomass is greater at lower availability. The increase in the intake rate of selected parts of palatable species during the green season is due to their high availability, but it could be more important if goats select for large bites in such a way as to maximize their instantaneous intake rate. As defined by Owen-Smith and Cooper [53], the term of palatability is applied to plant parts readily eaten when accessible by animals. Ungar and Noy-Meir [49] declared that herbivores tend to have this behavior when intake is limited by availability.

As observed during the summer and fall, goats tend to compensate for the low intake rate by extending daily grazing time, thus reducing the sensitivity of intake rate to the forage availability. Nevertheless, this strategy depends on the daily decision of herders and on the environmental stress imposed by browsing goats at specific times of the day usually allocated for other grazing activities, i.e., rumination and resting [49]. Herders tend the flock throughout grazing itineraries every day, crossing a mosaic of feeding stations. Herders observe their flock’s attitudes during grazing to evaluate initial hunger, intermediate disaffection, and signs of satiety. The herders’ strategy consists of interacting with spontaneous animal decisions to find requested forages and to meet their dietary requirements in a heterogeneous pasture [54]. However, the misinterpretation of satiety signs of goats could drive a wrong decision of the herder by reducing daily grazing time that would lead to a reduction in the daily intake rate. This situation is frequently observed when the flock is headed by another family member with limited herding experience. So the daily engagement of herders to other light agricultural activities are at the expense of time devoted to grazing goats.

During spring, goats exhibit preference and selectivity for *C. crispus, C. monspeliensis*, and *L. stoechas*, the species associated with large bite mass. *Cistus spp.* is known for a continuous vegetation growth that lasts 9 months from early fall until summer [55]. Spring is the flowering period for this group species when a high number and emergence of leaves and a high rate of shoot length are observed [55]. However, they contain a low level of nitrogen compared to winter because, in this growth season, this nutriment is retranslocated from leaves to new organs [55]. The nitrogen content of *Cistus spp*. is higher than 1%, equivalent to more than 6.25% of proteins [55,56], which is in the range of threshold level for efficient feed utilization that does not negatively affect feed intake [57]. The low content of nitrogen could mean a high content of nitrogen-free extract or soluble carbohydrates that reflect the high digestibility and nutritional quality of *Cistus spp.* as ruminant forage. Bruno-Soares et al. [56] reported for *C. salviifolius* leaves, a low content of condensed tannins (CT) from March to May compared to fall. The low content of CT and the high content of soluble carbohydrates could explain the high selectivity of *C. crispus* and *C. monspeliensis* during the green season. Compared to the current results, Mancilla Leytón et al. [58] reported that *L. stoechas* is more selected by goats during spring and also during summer. *L. stoechas* is characterized by the absence of physical defense [59] and offers high metabolizable energy [60], which could explain the goat preference for this species during spring that coincides with the flowering stage. The low selectivity of *A. unedo* and *E. arborea* during all seasons could be explained by their chemical composition and nutritional quality. *A. unedo* contains low and high levels of crude protein (CP) and CT, respectively [61]. As for *E. arborea*, it is characterized by low digestibility of dry [62] and organic matter [63], which means low nutritional quality. Also, this pastoral species contains a high concentration of CT [61].

During the dry season and fall, trees and some shrubs were more selected by goats despite their low availability because they represent evergreen forages with persistent leaves [64], even they are characterized by low proteins and high content of lignin and secondary compounds [65]. This statement was also reported by several authors in Mediterranean rangelands [14,16,30]. Particular high and positive preference was observed for *Q. suber* throughout the season of both years. Similarly, Manousidis et al. [14] reported high selectivity for *Q. frainetto* during all seasons in a Northern Mediterranean forest. *Q. suber* is an evergreen plant [66] with stem and leaf growth essentially in spring and with a low rate in the fall [65]. Cabiddu et al. [67] reported a high CP content in *Q. suber* leaves during spring and summer, which covers the maintenance requirement of goats, and could explain *Q. suber* preference. Gasmi-Boubaker and Kayouli [68] found a similar and stable CP content during all seasons with values higher than 8%, which makes *Q. suber* a stable nitrogen resource for goats in pastures. According to Oliveira et al. [69], nitrogen in leaves has a relative tendency to be higher during summer when the other pastoral species could contain low levels of proteins.

High selectivity of some plant species can also be explained by their seasonal spatial arrangement, which increases their opportunity to be selected. As reported by Wallis De Vries et al. [70], diet selection during grazing is more affected at the large scale by the spatial arrangement of the feeding stations and by the scale of patchiness, which impacts time and energy budgets of grazing animals in their search for more favorable feeding stations. Nevertheless, it should be remembered that the diet selection of goats is ultimately influenced by the herder’s decisions, who puts the herd under the constraint of time in different sectors and forces them to sometimes follow a specific grazing circuit. Herders take the animals to graze a sector of high palatable species according to their knowledge of the circuit [26] and the seasonal change of phenological states of plants [71]. 

Most optimal foraging models predict that behaviors trend towards maximizing the intake rate [72,73]. For goats, this trend is easier through their ability to switch rapidly between vegetation strata, mainly during the seasons of low forage availability [6]. During spring, it was noted that when forages are more available, goats avoided small patches and concentrated on the larger ones where foraging costs are low (low of switching movement). Consequently, goats spend greater time in each feeding station in comparison to the summer and fall. As observed during grazing, this duration depends on the degree of palatable plant presence and the number of goats grazing in the same feeding station. Similarly, it was reported that the optimal diet depends on the combination of the encounter rate of the feeding station and plant palatability [50].

### 4.3. Diet Diversity and Overlap

Diet diversity or niche breadth is directly affected by the proportion of plants in the diet. The diversity index was higher during the fall and summer of both studied years, probably due to the feeding strategy of goats, which visit many different feeding stations to meet their dietary requirements. Consequently, they included different plant species in their diet during these seasons to maximize the quantity of ingested forage, which could explain the extent of their dietary niche. El Aich et al. [19] also confirmed that goats consume a wider variety of plant species during the summer and fall, which enlarges their diet breadth. The green season is characterized by high feed offers and the appearance of some high palatable species [74], which are preferred by goats. Therefore, goats included in their diet only the high palatable species, which could explain the low diet diversity recorded during this season. As shown in the results, the diet composition of goats during spring was largely dominated by four plant species (*Cistus* spp. and *L. stoechas*, >83%; Table 3). El Aich et al. [19] also signaled a low diet diversity during early spring in the Argan forest. Diet diversity is probably influenced by the sequence of encounter rates with feeding stations of different profitability, which is dependent on the relationship between grazing tactic and spatial arrangement of plants.

The results show a high level of diet overlap between the same seasons of 2016 and 2017 (from 0.82 to 0.91). This high diet overlap could be explained by the similar selection of a mixture of plant species during the same seasons of the two studied years. The low diet similarity of spring with summer and fall seasons could be explained by the selection of different diets favored by the high availability and selection during the green season of distinct plant species such as *Cistus* spp. and *L. stoechas*.

## 5. Conclusions

The results emphasize the high goat preference for the woody species for which the level depends on grazing seasons. Despite their low availability, *Q. suber* contributed largely to the diet of goats across seasons. Diet selection was not necessarily correlated with the availability of each consumed plant species; it depended rather on the instantaneous foraging behavior of goats, which adapted their diet according to their energy intake requirements and plant species palatability. Despite the high variability of climate conditions in the Southern part of the Mediterranean region, this study confirms the high adaptability and plasticity of goats for the North Moroccan forest rangelands. This high dynamism and ability of goats to select woody species independently to the season and the year enables them to benefit from free feeding, thus allow herders to maintain their goats in a production system without feed supplementation costs. Overall, these findings are the first database about seasonal and year-to-year variations of foraging behavior of goats in Southern Mediterranean forest rangeland. These results could be used as the first guide about foraging strategies of grazing goats for future studies, decision-makers, and rangeland managers. 

Future research should consider the relationship between forage availability, diet quality, animal productivity, and relevance for current and possible emerging production systems, and the effect of climate change.

## Figures and Tables

**Table 1:** Forage availability (kg DM/ha), bite rate (bites/min), intake rate (g DM/min), and diet diversity (index) of goats browsing a Southern Mediterranean forest rangeland during 2016 and 2017.

Year	Season	Availability	BR ^1^	IR ^2^	Diet Diversity
2016	Spring	2064 ^A,3^	16.9 ^C^	4.41 ^A^	0.27 ^B^
	Summer	1289 ^B^	19.8 ^B^	3.21 ^B^	0.46 ^A^
	Fall	1018 ^C^	22.1^A^	3.32 ^B^	0.41 ^A^
	SEM ^4^	33.1	0.753	0.240	0.030
	*p*-Value (S)	<0.001	<0.001	<0.001	<0.001
2017	Spring	2590 ^A^	13.9 ^C^	5.10 ^A^	0.21 ^B^
	Summer	1670 ^B^	18.5 ^B^	4.03 ^B^	0.40 ^A^
	Fall	1328 ^C^	22.8 ^A^	4.25 ^B^	0.43 ^A^
	SEM	38.1	0.993	0.370	0.044
	*p*-Value (S)	<0.001	<0.001	<0.001	0.001
2016–2017					
Season (S)	*p*-Value	<0.001	<0.001	<0.001	<0.001
Year (Y)	*p*-Value	<0.001	<0.001	<0.001	<0.001
Y*S	*p*-Value	0.007	0.026	0.465	0.015

^1^ BR: bite rate; ^2^ IR: intake rate; ^3^ For the same year, means with different superscripts (A–C) within the column indicate significant differences (*p* < 0.05), ^4^ SEM: standard error of the mean.

**Table 2:** Bite mass (g DM) of plant species consumed by goats browsing a Southern Mediterranean forest rangeland during 2016 and 2017.

Item	2016					2017					*p*-Value (2016–2017)		
	Spring	Summer	Fall	SEM ^1^	*p*-Value	Spring	Summer	Fall	SEM	*p*-Value	S ^2^	Y ^3^	Y*S
*Arbutus unedo*	0.092 ^C,4,g,5^	0.190 ^A,b^	0.161 ^B,cd^	0.006	<0.001	0.099 ^B,d^	0.211 ^A,c^	0.203 ^A,c^	0.006	<0.001	<0.001	<0.001	<0.001
*Calicotome villosa*	0.040 ^C,h^	0.130 ^A,ef^	0.110 ^B,fg^	0.005	<0.001	0.093 ^C,de^	0.150 ^B,e^	0.170 ^A,de^	0.005	<0.001	<0.001	<0.001	<0.001
*Cistus crispus*	0.309 ^A,b^	0.088 ^B,hi^	0.051 ^B,i^	0.012	<0.001	0.405 ^A,a^	0.089 ^C,gh^	0.104 ^B,fg^	0.014	<0.001	<0.001	<0.001	<0.001
*Cistus monspeliensis*	0.339 ^A,a^	0.078 ^B,i^	0.040 ^B,ij^	0.014	<0.001	0.430 ^A,a^	0.078 ^B,h^	0.078 ^B,h^	0.015	<0.001	<0.001	<0.001	<0.001
*Cistus salviifolius*	0.289 ^A,bc^	0.060 ^B,j^	0.031 ^B,jk^	0.012	<0.001	0.370 ^A,b^	0.101 ^B,g^	0.089 ^B,gh^	0.013	<0.001	<0.001	<0.001	<0.001
*Erica arborea*	0.193 ^A,e^	0.168 ^B,c^	0.131 ^C,e^	0.007	<0.001	0.223 ^A,c^	0.101 ^C,g^	0.181 ^B,cde^	0.007	<0.001	<0.001	0.349	<0.001
Herbaceous	0.258 ^A,d^	0.081 ^C,i^	0.101 ^B,gh^	0.009	<0.001	0.411 ^A,a^	0.128 ^B,f^	0.112 ^B,fg^	0.015	<0.001	<0.001	<0.001	<0.001
*Lavandula stoechas*	0.279 ^A,cd^	0.052 ^B,j^	0.021 ^C,k^	0.012	<0.001	0.361 ^A,b^	0.089 ^B,gh^	0.079 ^C,h^	0.013	<0.001	<0.001	<0.001	<0.001
*Myrtus communis*	0.041 ^C,h^	0.195 ^A,b^	0.179 ^B,b^	0.008	<0.001	0.079 ^B,def^	0.280 ^A,b^	0.258 ^A,ab^	0.010	<0.001	<0.001	<0.001	<0.001
*Olea europaea*	0.028 ^C,h^	0.129 ^B,ef^	0.170 ^A,bc^	0.006	<0.001	0.050 ^C,g^	0.209 ^A,c^	0.188 ^B,cd^	0.007	<0.001	<0.001	<0.001	<0.001
*Phillyrea media*	0.029 ^C,h^	0.167 ^A,c^	0.150 ^B,d^	0.007	<0.001	0.050 ^C,g^	0.203 ^A,c^	0.176 ^B,cde^	0.008	<0.001	<0.001	<0.001	0.144
*Pistacia lentiscus*	0.049 ^C,h^	0.118 ^B,fg^	0.169 ^A,bc^	0.006	<0.001	0.070 ^C,efg^	0.260 ^A,b^	0.241 ^B,b^	0.009	<0.001	<0.001	<0.001	<0.001
*Quercus canariensis*	0.039 ^B,h^	0.139 ^A,de^	0.151 ^A,d^	0.006	<0.001	0.059 ^C,fg^	0.161 ^B,e^	0.189 ^A,cd^	0.006	<0.001	<0.001	<0.001	0.015
*Quercus ilex*	0.047 ^C,h^	0.152 ^A,cd^	0.122 ^B,ef^	0.006	<0.001	0.041 ^C,g^	0.182 ^A,d^	0.159 ^B,e^	0.009	<0.001	<0.001	<0.001	<0.001
*Quercus suber*	0.124 ^C,f^	0.239 ^A,a^	0.198 ^B,a^	0.006	<0.001	0.243 ^C,c^	0.341 ^A,a^	0.277 ^B,a^	0.009	<0.001	<0.001	<0.001	<0.001
*Rubus ulmifolius*	0.044 ^C,h^	0.104 ^A,gh^	0.089 ^B,h^	0.004	<0.001	0.042 ^C,g^	0.141 ^A,ef^	0.118 ^B,f^	0.009	<0.001	<0.001	<0.001	<0.001
SEM	0.002	0.003	0.002			0.002	0.004	0.002				
*p*-Value	<0.001	<0.001	<0.001			<0.001	<0.001	<0.001				

**^1^** SEM: standard error of the mean; **^2^** S: season; **^3^** Y: year; **^4^** Means with different capital letters (A–C) in the same row indicate significant differences (*p* < 0.05). **^5^** Means with different lowercase letters (a–k) in the same column indicate significant differences (*p* < 0.05).

**Table 3:** Diet composition (% of DM) of goats browsing a Southern Mediterranean forest rangeland during 2016 and 2017.

Item	2016					2017					*p*-Value (2016–2017)		
	Spring	Summer	Fall	SEM ^1^	*p*-Value	Spring	Summer	Fall	SEM	*p*-Value	S ^2^	Y ^3^	Y*S
*Arbutus unedo*	0.370 ^C,4^	12.9 ^A^	11.1 ^B^	0.431	<0.001	0.090 ^C^	8.37 ^B^	11.5 ^A^	0.373	<0.001	<0.001	0.026	<0.001
*Calicotome villosa*	0.020 ^C^	7.84 ^A^	4.32 ^B^	0.254	<0.001	0.010 ^C^	7.02 ^A^	2.44 ^B^	0.252	<0.001	<0.001	<0.001	<0.001
*Cistus crispus*	19.8 ^A^	0.170 ^C^	0.270 ^B^	0.657	<0.001	28.1 ^A^	0.500 ^B^	0.990 ^B^	0.932	<0.001	<0.001	<0.001	<0.001
*Cistus monspeliensis*	28.8 ^A^	0.170 ^B^	0.120 ^B^	0.932	<0.001	28.8 ^A^	0.300 ^B^	0.690 ^B^	0.960	<0.001	<0.001	0.502	0.758
*Cistus salviifolius*	17.6 ^A^	0.050 ^B^	0.070 ^B^	0.544	<0.001	15.8 ^A^	0.380 ^B^	0.360 ^B^	0.554	<0.001	<0.001	0.150	<0.001
*Erica arborea*	4.34 ^C^	11.1 ^A^	9.66 ^B^	0.283	<0.001	2.44 ^C^	9.16 ^B^	11.8 ^A^	0.374	<0.001	<0.001	0.082	<0.001
Herbaceous	7.14 ^A^	0.670 ^C^	1.99 ^B^	0.254	<0.001	7.54 ^A^	1.98 ^B^	1.20 ^B^	0.314	<0.001	<0.001	0.301	0.017
*Lavandula stoechas*	17.3 ^A^	0.490 ^B^	0.130 ^B^	0.562	<0.001	14.7 ^A^	0.270 ^B^	0.900 ^B^	0.564	<0.001	<0.001	0.050	<0.001
*Myrtus communis*	0.370 ^C^	14.3 ^B^	19.4 ^A^	0.610	<0.001	0.100 ^C^	14.4 ^B^	16.6 ^A^	0.622	<0.001	<0.001	0.033	0.016
*Olea europaea*	0.034 ^C^	6.72 ^A^	1.90 ^B^	0.221	<0.001	0.040 ^C^	6.93 ^A^	5.66 ^B^	0.291	<0.001	<0.001	<0.001	<0.001
*Phillyrea media*	0.200 ^B^	6.72 ^A^	6.50 ^A^	0.284	<0.001	0.050 ^C^	7.90 ^A^	4.38 ^B^	0.330	<0.001	<0.001	0.240	<0.001
*Pistacia lentiscus*	0.320 ^C^	7.96 ^B^	12.8 ^A^	0.403	<0.001	0.090 ^C^	15.4 ^B^	18.5 ^A^	0.625	<0.001	<0.001	<0.001	<0.001
*Quercus canariensis*	0.010 ^C^	2.98 ^B^	5.90 ^A^	0.394	<0.001	0.010 ^B^	2.21 ^A^	3.10 ^A^	0.214	<0.001	<0.001	0.004	0.015
*Quercus ilex*	0.120 ^B^	5.57 ^A^	4.83 ^A^	0.230	<0.001	0.020 ^B^	4.29 ^A^	4.15 ^A^	0.225	<0.001	<0.001	0.004	0.125
*Quercus suber*	3.57 ^C^	20.1 ^A^	17.8 ^B^	0.581	<0.001	2.19 ^B^	18.4 ^A^	16.2 ^A^	0.626	<0.001	<0.001	0.002	0.973
*Rubus ulmifolius*	0.010^B^	2.36 ^A^	3.36 ^A^	0.250	<0.001	0.010 ^C^	2.50 ^A^	1.58 ^B^	0.167	<0.001	<0.001	0.039	0.005

**^1^** SEM: standard error of the mean; **^2^** S: season; **^3^** Y: year; **^4^** Means with different capital letters (A–C) in the same row indicate significant differences (*p* < 0.05).

**Table 4:** Total bites (%) of goats browsing a Southern Mediterranean forest rangeland during 2016 and 2017.

Item	2016					2017					*p*-Value (2016–2017)			
	Spring	Summer	Fall	SEM ^1^	*p*-Value	Spring	Summer	Fall	SEM	*p*-Value		S ^2^	Y ^3^	Y*S
*Arbutus unedo*	1.07 ^B,4^	11.1 ^A^	10.5 ^A^	0.372	<0.001	0.342 ^C^	8.69 ^B^	11.8 ^A^	0.383	<0.001		<0.001	0.026	<0.001
*Calicotome villosa*	0.151 ^C^	9.80 ^A^	5.90 ^B^	0.322	<0.001	0.043 ^C^	10.2 ^A^	2.91 ^B^	0.356	<0.001		<0.001	<0.001	<0.001
*Cistus crispus*	16.8 ^A^	0.303 ^B^	0.792 ^B^	0.546	<0.001	26.5 ^B^	1.18 ^A^	2.00 ^A^	0.866	<0.001		<0.001	<0.001	<0.001
*Cistus monspeliensis*	22.2 ^A^	0.352 ^B^	0.444 ^B^	0.718	<0.001	25.4 ^A^	0.791 ^B^	1.74 ^AB^	0.839	<0.001		<0.001	<0.001	0.002
*Cistus salviifolius*	15.9 ^A^	0.131 ^B^	0.343 ^B^	0.512	<0.001	16.1 ^A^	0.82 ^B^	0.812 ^B^	0.558	<0.001		<0.001	0.070	0.818
*Erica arborea*	5.95 ^B^	10.8 ^A^	11.2 ^A^	0.284	<0.001	4.07 ^C^	10.5 ^B^	13.3 ^A^	0.427	<0.001		<0.001	0.931	<0.001
Herbaceous	7.21 ^A^	1.34 ^C^	2.99 ^B^	0.255	<0.001	6.95 ^A^	3.27 ^B^	2.21 ^B^	0.294	<0.001		<0.001	0.344	0.001
*Lavandula stoechas*	16.2 ^A^	1.60 ^B^	0.990 ^B^	0.506	<0.001	15.4 ^A^	0.642 ^B^	2.30 ^B^	0.565	<0.001		<0.001	0.648	0.020
*Myrtus communis*	2.39 ^C^	12.2 ^B^	16.4 ^A^	0.472	<0.001	0.494 ^C^	11.3 ^B^	13.0 ^A^	0.486	<0.001		<0.001	<0.001	0.035
*Olea europaea*	0.292 ^C^	8.39 ^A^	1.67 ^B^	0.278	<0.001	0.292 ^B^	7.22 ^A^	6.08 ^A^	0.309	<0.001		<0.001	<0.001	<0.001
*Phillyrea media*	1.68 ^B^	6.43 ^A^	6.53 ^A^	0.259	<0.001	0.344 ^C^	8.63 ^A^	4.94 ^B^	0.352	<0.001		<0.001	0.464	<0.001
*Pistacia lentiscus*	1.65 ^B^	10.8 ^A^	11.4 ^A^	0.375	<0.001	0.505 ^C^	12.9 ^B^	15.7 ^A^	0.528	<0.001		<0.001	<0.001	<0.001
*Quercus canariensis*	0.083 ^C^	3.46 ^B^	5.95 ^A^	0.403	<0.001	0.055 ^B^	2.93 ^A^	3.33 ^A^	0.258	<0.001		<0.001	0.016	0.038
*Quercus ilex*	0.581 ^B^	6.01 ^A^	6.07 ^A^	0.262	<0.001	0.111 ^B^	5.15 ^A^	5.26 ^A^	0.266	<0.001		<0.001	0.013	0.836
*Quercus suber*	7.79 ^B^	13.6 ^A^	13.5 ^A^	0.314	<0.001	3.38 ^B^	12.0 ^A^	11.9 ^A^	0.414	<0.001		<0.001	<0.001	0.005
*Rubus ulmifolius*	0.091 ^B^	3.73 ^A^	5.46 ^A^	0.367	<0.001	0.025 ^B^	3.78 ^A^	2.66 ^A^	0.245	<0.001		<0.001	0.015	0.003

**^1^** SEM: standard error of the mean; **^2^** S: season; **^3^** Y: year; **^4^** Means with different capital letters (A–C) in the same row indicate significant differences (*p* < 0.05).

**Table 5:** Selectivity index of plant species consumed by goats browsing a Southern Mediterranean forest rangeland during 2016 and 2017.

Item	2016					2017					*p*-Value (2016–2017)		
	Spring	Summer	Fall	SEM ^1^	*p*-Value	Spring	Summer	Fall	SEM	*p*-value	S ^2^	Y ^3^	Y*S
*Arbutus unedo*	−0.95 ^B,4^	−0.03 ^A^	−0.05 ^A^	0.03	0.003	−0.99 ^C^	−0.32 ^B^	−0.06 ^A^	0.05	<0.001	<0.001	<0.001	<0.001
*Calicotome villosa*	−0.96 ^C^	0.73 ^A^	0.47 ^B^	0.05	<0.001	−0.99 ^C^	0.60 ^A^	−0.002 ^B^	0.03	<0.001	<0.001	<0.001	<0.001
*Cistus crispus*	0.44 ^A^	−0.96 ^B^	−0.94 ^B^	0.05	0.004	0.49 ^A^	−0.91 ^C^	−0.85 ^B^	0.05	<0.001	<0.001	<0.001	0.045
*Cistus monspeliensis*	0.39 ^A^	−0.98 ^B^	−0.98 ^B^	0.04	0.008	0.36 ^A^	−0.97 ^C^	−0.92 ^B^	0.04	<0.001	<0.001	0.021	<0.001
*Cistus salviifolius*	−0.04 ^A^	−1.00 ^B^	−0.99 ^B^	0.03	0.005	−0.05 ^A^	−0.96 ^B^	−0.97 ^B^	0.04	0.006	<0.001	0.067	0.146
*Erica arborea*	−0.61 ^C^	−0.13 ^A^	−0.24 ^B^	0.02	<0.001	−0.79 ^C^	−0.33 ^B^	−0.18 ^A^	0.03	<0.001	<0.001	<0.001	<0.001
Herbaceous	−0.17 ^A^	−0.73 ^C^	−0.32 ^B^	0.03	<0.001	−0.43 ^A^	−0.58 ^B^	−0.58 ^B^	0.02	0.004	<0.001	<0.001	<0.001
*Lavandula stoechas*	0.72 ^A^	−0.70 ^B^	−0.91 ^C^	0.05	<0.001	0.36 ^A^	−0.90 ^C^	−0.71 ^B^	0.02	<0.001	<0.001	<0.001	<0.001
*Myrtus communis*	0.40 ^B^	1.00 ^A^	0.99 ^A^	0.03	<0.001	−0.66 ^C^	0.63 ^B^	0.97 ^A^	0.04	<0.001	<0.001	<0.001	<0.001
*Olea europaea*	−0.74 ^C^	0.99 ^A^	0.09 ^B^	0.06	<0.001	−0.71 ^C^	0.61 ^B^	0.91 ^A^	0.06	<0.001	<0.001	0.006	<0.001
*Phillyrea media*	0.03 ^C^	0.59 ^B^	0.89 ^A^	0.05	<0.001	−0.77 ^C^	0.55 ^B^	0.80 ^A^	0.06	<0.001	<0.001	<0.001	<0.001
*Pistacia lentiscus*	−0.61 ^B^	0.71 ^A^	0.76 ^A^	0.05	<0.001	−0.84 ^B^	0.83 ^A^	0.90 ^A^	0.06	0.002	<0.001	0.557	<0.001
*Quercus canariensis*	−0.98 ^C^	−0.04 ^B^	0.64 ^A^	0.06	<0.001	−0.98 ^C^	−0.26 ^B^	0.30 ^A^	0.06	<0.001	<0.001	0.002	0.081
*Quercus ilex*	−0.84 ^B^	0.54 ^A^	0.50 ^A^	0.06	0.007	−0.98 ^C^	0.25 ^B^	0.48 ^A^	0.06	<0.001	<0.001	0.005	0.107
*Quercus suber*	0.81 ^B^	1.00 ^A^	0.93 ^A^	0.01	0.003	0.01 ^B^	0.86 ^A^	0.99 ^A^	0.06	0.007	<0.001	<0.001	<0.001
*Rubus ulmifolius*	−0.86 ^B^	0.55 ^A^	0.67 ^A^	0.07	0.007	−0.98 ^B^	0.13 ^A^	0.08 ^A^	0.06	0.009	<0.001	<0.001	0.024

**^1^** SEM: standard error of the mean; **^2^** S: season; **^3^** Y: year; **^4^** Means with different capital letters (A–C) in the same row indicate significant differences (*p* < 0.05).

**Table 6:** Diet overlaps of goats browsing a Southern Mediterranean forest rangeland during 2016 and 2017.

Item		2016			2017		
		Spring	Summer	Fall	Spring	Summer	Fall
2016	Spring	-	0.10	0.09	0.91	0.10	0.12
	Summer	0.10	-	0.82	0.05	0.82	0.84
	Fall	0.09	0.82	-	0.05	0.77	0.86
2017	Spring	0.91	0.05	0.05	-	0.06	0.08
	Summer	0.10	0.82	0.77	0.06	-	0.83
	Fall	0.12	0.84	0.86	0.08	0.83	-
